# Community Recruitment of Asian, Latino and African American Older Adults With Depression Symptoms During COVID-19

**DOI:** 10.1093/geroni/igab046.1826

**Published:** 2021-12-17

**Authors:** Irene Falgas Bague, Ravali Mukthineni, Sahnah Lim, Aida Jimenez, Caroline Ferreira, Sheri Lapatin Markle, Margarita Alegria

**Affiliations:** 1 Massachusetts General Hospital, Harvard Medical School, Boston, Massachusetts, United States; 2 Disparities Research Unit. Massachusetts General Hospital, Boston, Massachusetts, United States; 3 New York University, New York, Massachusetts, United States; 4 Department of Psychology, University Of Puerto Rico - Rio Piedras/San Juan, Massachusetts, United States

## Abstract

Recruitment and engagement of racial/ethnic minority older adults in clinical trials is crucial to expand implementation of evidence-based interventions for disability prevention. Public Health measures to counteract COVID-19 pandemic have increased the challenges on reaching this population. This study seeks to comprehensively evaluate a set of recruitment strategies to enroll Latino, Asian and African American older adults with symptoms of depression and anxiety during the first year of a randomized clinical trial. A partnership of three academic sites across the U.S. (NYC, MA and PR) involving several collaborations with community agencies recruited racial/ethnic minority older adults using different strategies involving bilingual interviewers calling from hospital research dataset and community agencies’ list of clients, referrals from primary care providers or psychotherapy waitlist. In this presentation we will report various recruitment and retention data including individual and organizational predictors of successful recruitment as well as challenges across all three sites.

